# H_2_O_2_ Metabolism in Normal Thyroid Cells and in Thyroid Tumorigenesis: Focus on NADPH Oxidases

**DOI:** 10.3390/antiox8050126

**Published:** 2019-05-10

**Authors:** Ildiko Szanto, Marc Pusztaszeri, Maria Mavromati

**Affiliations:** 1 Department of Internal Medicine, Division of Endocrinology, Diabetes, Hypertension and Nutrition, Geneva University Hospitals, 1205 Geneva, Switzerland; Maria.Mavromati@hcuge.ch; 2 Diabetes Centre, Faculty of Medicine, University of Geneva, 1211 Geneva, Switzerland; 3 Department of Pathology, Jewish General Hospital and McGill University, Montréal, Québec, QC H3T 1E2, Canada; marc.pusztaszeri@mcgill.ca

**Keywords:** NADPH oxidase, NOX4, dual oxidase, DUOX2, Thyroid, redox

## Abstract

Thyroid hormone synthesis requires adequate hydrogen peroxide (H_2_O_2_) production that is utilized as an oxidative agent during the synthesis of thyroxin (T4) and triiodothyronine (T3). Thyroid H_2_O_2_ is generated by a member of the family of NADPH oxidase enzymes (NOX-es), termed dual oxidase 2 (DUOX2). NOX/DUOX enzymes produce reactive oxygen species (ROS) as their unique enzymatic activity in a timely and spatially regulated manner and therefore, are important regulators of diverse physiological processes. By contrast, dysfunctional NOX/DUOX-derived ROS production is associated with pathological conditions. Inappropriate DUOX2-generated H_2_O_2_ production results in thyroid hypofunction in rodent models. Recent studies also indicate that ROS improperly released by NOX4, another member of the NOX family, are involved in thyroid carcinogenesis. This review focuses on the current knowledge concerning the redox regulation of thyroid hormonogenesis and cancer development with a specific emphasis on the NOX and DUOX enzymes in these processes.

## 1. Introduction

The synthesis of thyroid hormones is a complex, multistep process that encompasses several redox reactions that utilize hydrogen peroxide (H_2_O_2_) as an oxidative agent. Hydrogen peroxide is contained within the lumen of the thyroid follicles which are considered as the functional units of the thyroid gland where hormone synthesis, storage and release take place. The follicles are formed by a monolayer of polarized epithelial cells, termed thyrocytes, that surround the central lumen of the follicle. The lumen is delimited by the apical surface of thyrocytes that are connected to each other by tight junctions preventing “leaking” of the lumen content into the extra-follicular space. The outer side of the follicle is sealed by the basolateral plasma membranes of thyrocytes. The lumen is filled by a protein-rich substance termed “colloid”, mainly consisting of thyroglobulin (TG), a 660kDa thyroid-specific protein. TG accounts for approximatively 50% of the protein content of the thyroid gland and serves as the precursor of thyroid hormones. Thyroid hormone synthesis requires the oxidative iodination of specific tyrosine residues of TG, a process termed “iodide organification”. Oxidative iodination is catalyzed by the enzyme thyroid peroxidase (TPO). This process is a key step in thyroid hormonogenesis requiring an appropriate amount of H_2_O_2_ for oxidation. The first study that highlighted this critical role was published in 1975 and described diminished H_2_O_2_ generation in human thyroid nodules with defective iodide organification [1]. The source of luminal H_2_O_2_ has later been characterized in isolated porcine thyroid follicles as a thyrocyte apical membrane enzyme that utilizes NADPH as electron donor and requires calcium for its activation [2]. Subsequent biochemical studies identified the thyrocyte H_2_O_2_ generating enzymes as members of the NADPH oxidase (NOX) family [3,4]. The proper identities of the enzymes were finally established in 1999-2000 by protein purification and sequencing as well as by thyroid cDNA library screening. The enzymes were considered as thyroid-specific thus, were named thyroid oxidases (THOX) [5,6,7]. Subsequently, the proteins were identified in other tissues and their names were modified for dual oxidase 1 and 2 (DUOX1/2) to reflect their dual oxidase and peroxidase activities [8]. The major source of H_2_O_2_ in the thyroid follicle is the isoform DUOX2 [5,6]. In contrast to DUOX2-generated physiological reactive oxygen species (ROS) production, pathologically elevated H_2_O_2_ levels are linked to thyroid carcinogenesis resulting from enhanced mitogenic receptor signals or oncogene activation [9,10,11]. In this respect, recently another NOX isoform, NOX4, has been identified as a potential source of harmful ROS [12,13]. 

This review provides a summary of current knowledge about the redox biology of the thyroid with a specific emphasis concerning the roles of NOX/DUOX enzymes in thyroid hormone biosynthesis and cancer.

## 2. Redox Enzymes in Thyroid Hormone Synthesis

The two main enzymes involved in the redox steps of thyroid hormone synthesis are TPO and DUOX2. TPO catalyzes iodination of TG tyrosine residues and subsequent iodotyrosine chain coupling, while DUOX2 supplies the H_2_O_2_ required by TPO as an electron acceptor in these reactions.

### 2.1. Thyroid Peroxidase (TPO)

Thyroid peroxidase is a thyroid-specific glycosylated hemoprotein that plays a key role in thyroid hormone synthesis. Thyroid hormones contain iodine that is absorbed in the small intestine, transported in the circulation and is taken up by thyrocytes via a Na/I symporter (NIS) localized in their basolateral plasma membrane. Iodine will then be transferred into the follicle lumen and oxidized by TPO employing H_2_O_2_ as oxidizing agent. TPO is a transmembrane protein expressed on the apical site of thyrocytes with its heme-containing pocket protruding into the lumen of the follicle [14]. The heme-pocket, the active center of TPO, is redox-sensitive and essential for activation of the enzyme [15,16]. TPO activity is mainly regulated by TSH, and also with oxidized iodine that acts as a negative regulator of TPO [17,18]. Oxidized iodine (iodinium or hypo-iodite) is chemically highly reactive allowing its subsequent incorporation in tyrosine residues of TG by TPO. The resulting iodotyrosine residues can then be coupled within the TG molecule to yield 3,5,3′,5′-tetraiodothyronine (T4/thyroxin), or 3,5,3′- triiodothyronine (T3). T4 and T3 will remain bound to TG and stored in the follicle until hormone liberation is required [4]. Upon appropriate stimuli for hormone release, TG is endocytosed by the thyrocytes and undergoes a proteolytic process by fusing with a lysosome. Proteolysis of TG results in the release of T3 and T4 from the prohormone. T4/T3 are ultimately transported into the bloodstream through diverse thyrocyte basolateral membrane transporters. 

### 2.2. Dual Oxidases (DUOX1 and DUOX2) 

**Figure 1:**
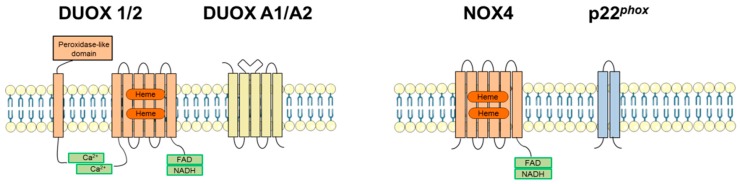
The structures of dual oxidases 1/2 (DUOX1/2) and NADPH oxidase 4 (NOX4). DUOX1 and 2 form intramembrane complexes through intermolecular disulphide bridges with dual oxidase activator or dual oxidase maturation factor 1 and 2 (DUOXA1 and 2, respectively). DUOXA1/2 are necessary for DUOX1/2 membrane translocation and enzymatic activity. DUOX1/2 are activated by Ca^2+^ through cytoplasmic calcium-binding EF-hand domains. DUOX1/2 also possess an extra N-terminal peroxidase-like domain. NOX4 forms a heterodimer with the membrane-anchored p22*^phox^* that is necessary and sufficient to support NOX4 constitutive ROS production. FAD: flavin adenine dinucleotide (FAD)-binding domain; NADPH: Dihydronicotinamide-adenine dinucleotide phosphate (NADPH)-binding domain; Ca^2+:^ Calcium-binding EF hand regions; Heme: heme-binding histidine residues.

DUOX1 and DUOX2 belong to the NADPH oxidase family that comprises of seven members: five NOXes (NOX1-5) and the two dual oxidases: DUOX1 and 2. NOX family enzymes are membrane proteins with two heme-binding regions, and a cytoplasmic C-terminus chain that encompasses binding sites for flavin adenine dinucleotide (FAD) and NADPH (reviewed in [19,20]). The isoforms NOX1, 2, 3 and 4 form heterodimers with the membrane-anchored subunit p22*^phox^* which is essential for the activation of the isoforms NOX1, NOX2 and NOX3. Indeed, NOX1, NOX2 and NOX3 require membrane translocation of diverse cytosolic organizer (p47*^phox^*/NOXO1) and activator (p67*^phox^*/NOXA) factors for active oxidase assembly, and p22*^phox^* plays a crucial role in this process by docking p47*^phox^*/NOXO1 [21]. Contrary to NOX1,-2 and -3, NOX4 does not need recruitment of other subunits for its activation but produces H_2_O_2_ in a continuous manner [22,23,24]. The role of p22*^phox^* in NOX4 regulation is currently incompletely understood. Co-transfection studies with NOX4 and p22*^phox^* have suggested that p22*^phox^* might be required for NOX4 stability, and/or maturation/localization [21,25,26]. Activation of the isoform NOX5 and the two DUOX-es are achieved through Ca^2+^ binding by EF-hand motives contained in their N-terminal cytoplasmic segments [27,28]. DUOX enzymes are distinguished from all other NOX-es by the presence of a seventh transmembrane domain and a unique N-terminal peroxidase ectodomain that displays 40% homology to TPO [29]. As a result, superoxide (O_2_•^−^) produced by DUOX enzymes is directly dismutated into H_2_O_2_ [7,30]. DUOX1/2 activation requires association with the accessory proteins DUOXA1 and DUOXA2 (see in following chapter for more details) [31,32,33]. The schematic structure of DUOX1/2 enzymes is depicted in comparison to NOX4, the other thyroid-relevant NOX isoform (see chapter 3.3) in Figure 1. 

**Figure 2:**
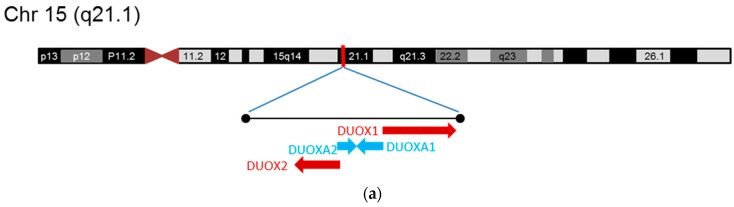

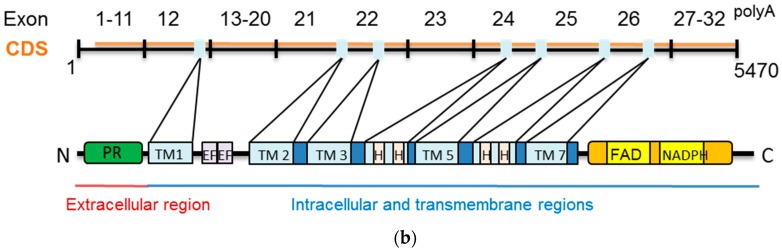
(**a**) Chromosomal organization of the DUOX1/2 and DUOXA1/2 genes. (**b**) Exon/intron and mRNA structure of DUOX2. CDS: coding sequence; PR: peroxidase domain; TM1–7: Transmembrane domains 1–7; EF: Ca^2+^-binding EF hand domains; FAD: flavin adenine dinucleotide-binding domain; NADPH: Dihydronicotinamide-adenine dinucleotide phosphate-binding domain; H: heme-binding histidine residues.

DUOX1 and DUOX2 are high molecular weight proteins (177kDa and 175kDa, respectively) that share significant sequence similarity with 77% identical amino acid sequences [5,6]. The *duox1* and *duox2* genes are located at the same locus on chromosome 15q21.1 but with an opposite orientation. The chromosomal organization of DUOX1/2 and DUOXA1/2 is shown in Figure 2a along with the exon-structure of DUOX2 in Figure 2b. Both DUOX1 and 2 are expressed in the thyroid, though the expression level of DUOX2 is five-fold higher compared to DUOX1 [7,30,34]. In addition to the thyroid, DUOX1 mRNA has been detected in airway and tongue epithelia, cerebellum and testis, while DUOX2 was observed in the respiratory and gastrointestinal tracts as well as in salivary and rectal glands, uterus, gallbladder and islets of Langerhans in the pancreas [7,30,35,36,37,38]. In airways, DUOX2 is implicated in innate immunity inflammatory response [39,40]. The physiological relevance of DUOX2 in other extrathyroidal tissues is currently not fully discovered. 

**Figure 3:**
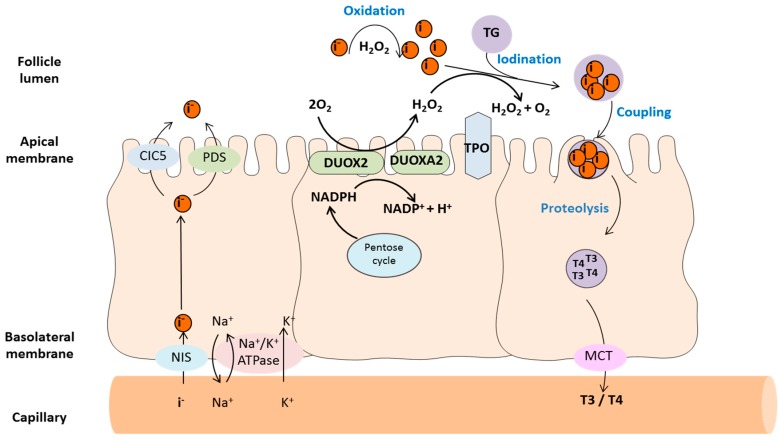
Thyroid hormone synthesis. Thyroid hormone synthesis follows a strict spatial and temporal schedule. Thyroid hormones (T4 and T3) are synthetized within the thyroid-specific protein thyroglobulin (TG) and they remain bound to it until the protein is degraded and T4/T3 are released into the circulation. Thyroglobulin is produced by the thyrocytes and secreted into the follicular lumen. The synthesis of T3/T4 requires iodination of the tyrosin residues of TG which occurs in the follicule lumen in a multistep process. The iodination consists of (1) iodide oxidation, (2) tyrosyl radical oxidation, (3) thyroglobulin iodination (iodine organification) and (4) intramolecular coupling of iodotyrosines to form T4 and T3. T4/T3 are then released into the capillary system by monocarboxylate transporters (MCT). Iodine is transported into the thyrocytes by the sodium/iodide symporter (NIS) located in their basolateral plasma membrane. The Na^+^ gradient providing the energy for this transport is maintained by the Na^+^/K^+^-ATPase. Iodine is then moved from the cytosol into the follicule lumen by the voltage gated chlorine channel CIC5 and the sodium-independent chloride-iodide transporter termed pendrin (PDS). The concentrated iodide is then rapidly oxidized by thyroid peroxidase (TPO) using H_2_O_2_ supplied by DUOX2 employing NADPH as electron donor. NADPH is mainly derived from the pentose cycle of glycolysis. NIS: sodium/iodide symporter; PDS: pendrin; MCT: monocarboxylate transporters; TG: Thyroglobulin; TPO: Thyroid peroxidase; T4: tetraiodothyronine/ L-thyroxine; T3: L-triiodothyronine.

During their maturation process, DUOX1/2 enzymes undergo N-glycosylation in the endoplasmic reticulum (ER) and the Golgi and are converted from a 180 kDa high mannose glycosylated (immature) form localized in the ER into a 190 kDa fully glycosylated (mature) form that is present at the luminal plasma membrane of thyrocytes [32,41]. The ER-to-Golgi transport and translocation to plasma-membrane are dependent on the co-expression of DUOX maturation factors, termed DUOXA1 and DUOXA2 that form heterodimers with DUOX1 and DUOX2, respectively. Associations between DUOX and DUOXA proteins are indispensable for DUOX H_2_O_2_ production [31,32,33]. In turn, DUOXA2 stability and function requires oxidative modification by DUOX2 [42]. Inaccurately paired (DUOX1-DUOXA2 or DUOX2-DUOXA1) dimers are functional but show hampered superoxide production compared to the appropriately paired dimers [32,33]. The co-expression of *duox1* and *duox2* with *duoxa1* and *duoxa2* genes is sustained by a specific genomic organization. In fact, *duoxa1* and *duoxa2* are located between the *duox1 and duox2* genes and their mRNA expression is regulated by the bidirectional promoter of *duox1* and *duox2* [43,44,45]. DUOXA1 and DUOXA2 are present in highest amounts in the thyroid gland but lower expression levels have also been detected in human respiratory epithelial cells (DUOXA1) and in the salivary gland (DUOXA2) [43,45]. The coordinated role of DUOX2 and TPO in the redox processes of thyroid hormonogenesis is summarized in Figure 3. 

In line with their thyroid modulatory function DUOX1/2 and DUOXA1/2 are regulated by diverse factors that affect thyroid hormone synthesis. Transcription of *duox2* mRNA is upregulated by the thyroid stimulating hormone (TSH) through cAMP-mediated intracellular signal transmission in dog and pig thyrocytes [5,6]. In rat thyroid cells, *duox2* and *duoxa2* mRNAs are suppressed by TG providing an autoregulatory mechanism to control thyroid hormone synthesis [46]. In contrast to animal thyrocytes, the mechanism of regulation of *duox2* transcription in human thyroid is currently unknown. Next to its transcriptional enhancing effect, TSH also increases enzymatic activity of both DUOX1 and DUOX2 albeit through different intracellular signaling pathways. Indeed, DUOX1 is activated by Protein kinase A through Gs-mediated signaling. By contrast DUOX2 is activated by Protein Kinase C through the Gq-phospholipase C (PLC) pathway [28]. In addition, DUOX activity and its association with TPO are also upregulated by a rise in intracellular H_2_O_2_ concentrations [29,47,48]. DUOX2-mediated H_2_O_2_ production is inhibited by excess iodine leading to decreased TPO activity and reduced incorporation of iodine into TG [17,47,49,50]. This inhibitory effect of iodine on its own organification was already described in 1948 and was named the “Wolff-Chaikoff effect” after the authors of the original paper [51]. In addition to these classical regulators, recent investigations identified the TH2 cytokines IL-4 and IL-13 as transcriptional activators of *duox2* and *duoxa2* genes in the respiratory tract and in the thyroid [52,53]. In line with these data, mice with thyroid-specific transgenic overexpression of IL-4 displayed enhanced expression of DUOX1 and pendrin [54]. Thyroid autoimmune diseases, most notably Hashimoto thyroiditis (HT) and Graves’ disease are characterized by alterations in TH1/TH2 cytokine profiles and the presence of increased ROS levels [55,56]. However, current knowledge does not support a direct link between DUOX enzymes, an imbalance in TH1/TH2 cytokines and these autoimmune thyroid pathologies [57]. The pathophysiological relevance of TH2-mediated regulation of *duox1/2* transcription in human thyroid (if any) is yet to be established.

## 3. NADPH Oxidases in Thyroid Pathologies

### 3.1. DUOX1/2 in Thyroid Dyshormonogenesis

Insufficient DUOX2-derived H_2_O_2_ was related to thyroid dyshormonogenesis both in vitro and in murine models with genetic ablation or spontaneous mutations within the *duox2* or *duoxa* genes [58,59,60,61,62,63]. A recent study found that in Zebrafish mutations of a single *duox* gene led to a phenotype associated hypothyroidism (growth retardation, goiter and infertility) and this phenotype could be reversed by T4 administration [64]. In humans however, the relationship between congenital hypothyroidism (CH) and DUOX2 functionality is more complex. The first case linking a defect in thyroid NADPH oxidase Ca^2+^-dependent H_2_O_2_ generation to goiter and hypothyroidism in two siblings was reported in 2001 [65]. The following year, inactivating mutations in DUOX2 (at that time still named THOX2) were identified in hypothyroidic patients with defective iodine organification [60]. Subsequent studies reported transient CH in patients with one allele mutations [60,66,67,68,69], and permanent CH in patients with homozygous mutations in the *duox2* or *duoxa2* genes [70,71,72,73]. However, later studies indicated that the severity of hormonogenesis deficiency is not directly proportional to DUOX2/DUOXA2 dysfunction (reviewed in detail in [59,60,74]). Indeed, in subsequent investigations, mono- and diallelic DUOX2 mutations were equally found in transient and permanent CH cases (reviewed in [59,74,75]). These data were confirmed by another recent study employing next generation sequencing which identified essentially monoallelic mutations in the *tpo* (30% of cases) and *duox2* (25% of cases) genes in patients with severe CH [76]. *Duox2* missense mutations that prevent DUOX2-DUOXA2 interaction and, thus result in DUOX2 retention and premature degradation, were also described in hypothyroid patients [43,66,67,68]. Taken together, these studies indicate the necessity for appropriate DUOX2-mediated H_2_O_2_ production in healthy thyroid function in humans though the exact relationship between the extent of redox imbalance and thyroid hypofunction is currently unclear.

In contrast to DUOX2, the role of DUOX1 in thyroid physiology remains largely unknown. DUOX1 knock-out mice do not present altered thyroid hormone levels and in humans no DUOX1 mutation in relation to thyroid dysfunction has been reported arguing against a significant role for the DUOX1 isoform in thyroid physiopathology [51,74,77]. 

### 3.2. DUOX1/2 in Thyroid Tumorigenesis

In contrast to other endocrine organs, the thyroid gland has a high tumor frequency rate despite of the very slow proliferation of thyroid cells [78,79,80]. Tumorigenesis in diverse organs has been linked to unbridled ROS production and several NOX family members have been implicated in this process [20,81]. The role of DUOX enzymes in tumorigenesis is ambiguous and far from completely uncovered. Epigenetic silencing of DUOX1 expression was reported in lung and hepatocellular carcinomas suggesting a possible tumor suppressing role for DUOX1 [45,82]. Other studies reached quite the opposite conclusion showing elevated expression of DUOX proteins in colon, prostate and breast cancer cells implying a tumorigenic role [27,83]. The conflicting anti- and pro-tumor effects of DUOX-derived H_2_O_2_ likely reflect the concentration-dependent double actions of ROS. Indeed, in physiological concentrations ROS exert mitogenic actions thus, promote tumor cell proliferation, but in case of excess intracellular accumulation they can provoke growth arrest and apoptosis thus, contribute to tumor suppression [84]*.* Concerning thyroid malignancies, elevated DUOX1 mRNA levels were reported in papillary thyroid carcinomas (PTC) and follicular adenomas of patients with a history of childhood radiation [85]. Overexpression of DUOX1 in radio-induced thyroid tumors suggests that DUOX1 may contribute to chronic oxidative stress and thus, can promote genomic instability and tumorigenesis. Indeed, it has been shown that H_2_O_2_ is able to cause *RET/PTC1* rearrangement which are frequently found in radiation-induced PTCs [80]. In primary human thyrocytes *in vitro*, siRNA-mediated knock-down of *duox1* resulted in significant reduction in radio-induced DNA damage [85]. The relationship between DUOX1 and radiation-related thyroid cancers in humans in vivo is not yet established as high level of DUOX1 protein was also observed in some sporadic thyroid tumors and a study comparing sporadic and radiation-induced PTCs found no difference between their respective *duox1* mRNA levels [85,86]. Therefore, the in vivo cause-effect relationship between *duox1* expression levels and thyroid cancer, in particular radiation-related PTCs remains to be elucidated. 

In contrast to its physiological function, the role of DUOX2 in thyroid cancer initiation and/or progression is much less understood (reviewed in [27]). The prevalent physiologic function of DUOX2 in the thyroid is suggested by data demonstrating unchanged or marginally diminished DUOX2 expression in differentiated thyroid carcinomas [87,88].

### 3.3. NADPH Oxidase 4 (NOX4)

NOX4 was initially cloned from kidney and was referred to as renal oxidase (Renox), however it was later shown to be expressed at lower levels in most tissues and cell types, including the thyroid gland [89,90,91,92,93]. NOX4 is anchored in plasma and intracellular membranes through interaction with the membrane-bound subunit p22*^phox^* and this interaction is necessary and sufficient for its constitutive ROS production [21]. Interestingly, electrons passing through NOX4 directly generate H_2_O_2_ with only 10% resulting in superoxide production [94]. This is an intrinsic feature of NOX4 involving its E-loop protruding into the extracellular space [95]. Concerning its cellular localization, NOX4 protein has been observed in diverse intracellular organelles, namely in the ER and perinuclear space, in proximity of focal adhesions, in the mitochondria and the nucleus [96,97,98,99,100]. Contrary to other NOX family enzymes that need stimulatory signals to induce their ROS production, NOX4 generates ROS in a constitutive manner and its activity is regulated mainly at the transcriptional level [8,24,101,102]. In addition, in vascular smooth muscle cells, NOX4 activity is also enhanced by an interaction with DNA polymerase-δ-interacting protein 2 (POLDIP2), and this interaction is an important component in the modulation of F-actin oxidation, focal adhesion maturation and cell adhesion [97,103]. 

In the thyroid, NOX4 has been recently detected by immunohistochemical investigations in the cytoplasm of both human and rat thyrocytes and in the plasma membrane of rat thyrocytes [104,105,106]. The function of NOX4 in physiological thyroid hormonogenesis is currently unknown. Mice with genetic ablation of NOX4 do not present any obvious thyroid phenotype though their thyroid function has not yet been evaluated in a targeted fashion [107]. One very recent study indicated enhanced NOX4 expression and ROS production as a link between goiter size and NIS regulation in Wistar rats [108]. Oglio et al. suggested a potential role for NOX4-derived ROS in iodine-induced autoregulatory mechanisms evidenced by the lack of inhibitory effect of iodine on thyroid-specific genes after NOX4 silencing in differentiated rat thyroid cells in vitro [109]. Taken together, these very recent data indicate the need of further investigations to elucidate the potential role of NOX4 in physiological thyroid hormone synthesis and iodine-related regulation.

#### NOX4 in Thyroid Tumorigenesis

While little is known about its physiological role in the thyroid, NOX4 has been more intensively investigated in thyroid tumorigenesis. Along this line, recent studies indicated a link between elevated NOX4 mRNA levels and thyroid dedifferentiation [13,105]. Supporting these data, TSH and insulin suppress NOX4, and elevate the expression levels of TPO and the thyroid-specific transcription factors Pax8 and TTF-2 in differentiated rat thyroid cells *in vitro.* On the contrary, NOX4 silencing increases expression of these genes [109]. These data indicate that elevated NOX4 transcription and thus, increased NOX4-derived H_2_O_2_ production, are linked to dysregulated thyroid differentiation status. Elevated NOX4 expression is a hallmark of several cancer types. In this respect, diverse cell type-specific intracellular signaling pathways related to the carcinogenic effect of NOX4-derived ROS have been identified [110,111]. In fibroblasts, the cancerogenic action of NOX4 has been linked to redox inactivation of the tumor suppressor p53 substantiating enhanced cell cycle progression [112]. Another NOX4-related intracellular pathway is the inactivation of endoplasmic reticulum resident protein phosphatase 1 (PP1) [113]. NOX4-mediated suppression of PP1 activity enhanced cell survival under ischemic conditions in cardiomyocytes and terminated epidermal growth factor (EGF) receptor signaling in vascular endothelial cells [114,115]. 

Concerning thyroid pathology in particular, an increasing number of studies described elevated NOX4 and p22*^phox^* expressions in thyroid nodules and cancer tissues [12,13,105,116]. In line with a cancerogenic role, in vitro knockdown of NOX4 in cancerous thyroid follicular cells counteracted iodolactone-induced ROS production and apoptosis [46,88,117]. NOX4 has also been demonstrated to play a key role in transforming growth factor beta 1 (TGF-β1) signal transmission in many cell types, both in vitro and in vivo [91,118,119,120,121]. NOX4 is unique in being induced by TGF-β. TGF-β is synthetized by thyrocytes and inhibits thyroid function and growth in differentiated thyroid cells [122,123,124]. Contrary to differentiated thyrocytes, undifferentiated cells of thyroid carcinomas are resistant to TGF-β anti-proliferative effects [125]. Data obtained from human thyroid follicular carcinoma cells implicated NOX4 as one of the mediators of this TGF-β resistance as siRNA-mediated silencing of NOX4 countered TGF-β inhibitory effect on cell proliferation [126]. 

The most common type of human thyroid malignancies is PTC, a well-differentiated type of cancer that usually carries a very good prognosis [127]. The propensity of the tumor cells to accumulate iodide, which is mediated by the thyroid sodium/iodide symporter (NIS), is usually retained in PTC which is clinically highly relevant because it enables treatment of residual or recurrent cancer following thyroidectomy with radioactive iodine. Approximately 40–60% of conventional PTCs carry the oncogenic V600E mutation of the serine/threonine phosphatase BRAF (BRAF^V600E^ tumors) resulting in constitutive activation of mitogen-activated protein kinase (MAPK) that results in uncontrolled proliferation [128,129,130]. An important feature of BRAF^V600E^-mutated PTC is its association with resistance to radioiodine treatment due to diminished expression of NIS following histone deacetylation of its promoter [131,132]. The tall cell and the hobnail variants of PTC, which are aggressive variants of PTC characterized by more extensive disease, a higher rate of recurrence, and decreased survival, carry the BRAF^V600E^ mutation in up to 90% and 70–80% of cases, respectively [133]. A recent study elucidated a crucial role for NOX4 in BRAF^V600E^ mutation related carcinogenesis by demonstrating that the BRAF^V600E^ oncogene induces NOX4 expression through the TGF-β/Smad3 pathway along with the downregulation of NIS expression, and the promotion of cell migration, invasiveness and epithelial-mesenchymal transition [13,86]. In line with these observations, genomic analysis of human BRAF^V600E^ thyroid tumors found inverse correlation between NOX4 expression and thyroid differentiation [27]. Furthermore, TP53 mutations are highly prevalent in both anaplastic thyroid carcinomas (ATC) and poorly differentiated thyroid carcinomas (PDTC), which have a higher mutation burden than PTC from which they can arise. This might be related to an increase in NOX4-induced DNA damage via ROS production, as mutant p53 proteins were also found to enhance NOX4 expression in both TGF-β-dependent and TGF-β-independent processes in human breast and lung epithelial cells [134].

Another study highlighted a different role for NOX4 in thyroid carcinogenesis by demonstrating that NOX4-related ROS production is an essential component in the metabolic adaptation of PTC cells [135]. Malignant tumors are hypoxic due to a relatively lower capillarization rate compared to their unbridled proliferation [136]. Hypoxia leads to the stabilization and nuclear accumulation of hypoxia-inducible factors (HIFs) triggering a complex transcription response to enable adaptation to diminished oxygen concentrations [137]. One of the adaptive mechanisms is shifting cellular glucose metabolism towards glycolysis, as opposed to oxidative phosphorylation (OXPHOS), that is typical of non-tumor cells and this shift was also observed in certain PTC cell lines [138,139]. This adaptive metabolic shift (commonly referred to as the Warburg effect) allows production of biomolecules (nucleotides, membrane lipids) to support enhanced mitotic activity at the cost of maintaining cellular energy production in a less efficient way [140]. Importantly, metabolic pathways linked to the Warburg effect have been suggested to modulate intracellular signaling by effecting cellular redox homeostasis. Two examples are the pentose phosphate shunt that generates NADPH and the de novo serine metabolism that generates both NADPH and glutathione [141,142]. NADPH and glutathione are important in the modulation of cellular redox balance and decrease in their cellular concentrations favors the onset of oxidative stress [143]. Elevated ROS levels along with decreased antioxidant enzymes have been reported in thyroid cancer specimen suggesting the onset of oxidative stress in these tissues [144]. It has been suggested that increased levels of ROS may contribute to PTC development in patients with Hashimoto thyroiditis, the most common form of chronic thyroiditis [145]. 

**Figure 4:**
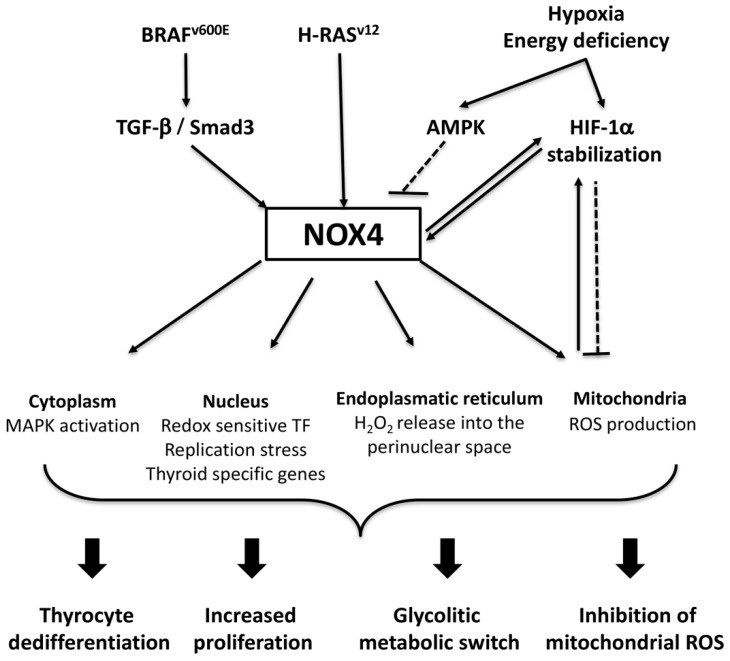
NOX4-related mechanisms in thyroid carcinogenesis. Mutations in the proto-oncogene BRAF increase NOX4 expression through the TGF-β/Smad3 pathway leading to constitutive MAPK activation and a decrease in thyroid-specific genes. H-RAS oncogenic mutations increase NOX4-mediated H_2_O_2_ production and thyrocyte survival. Energy deficiency induces AMPK which in turn inhibits NOX4 activity. Hypoxia leads to the stabilization of the transcription factor HIF-1α. HIF-1α induces NOX4 expression and reduces mitochondrial ROS production. ROS produced by NOX4 and the mitochondria increases HIF-1α activity providing a feed-back loop to mitigate ROS-induced cell damage. Mitochondrial ROS also activates AMPK (not shown on the scheme). NOX4-derived H_2_O_2_ exerts its effects in direct and indirect ways in various cell compartments. Taken together, these different pathways interact and mediate different aspects of thyroid carcinogenesis, most notably thyrocyte dedifferentiation, enhanced cell proliferation and survival, a metabolic switch towards glycolysis and an inhibition of mitochondrial ROS.

NOX4 is a target of HIF-1α and is a critical player in the maintenance of hypoxia-related ROS production and proliferation [146]. In PTC cells NOX4 knockdown diminished proliferation, reduced mitochondrial ROS production and decreased HIF-1α stabilization [135]. In addition, NOX4 was essential for the metabolic switch towards anaerobic glycolysis, an important component in the maintenance of cell proliferation of tumorous cells [135]. Thyroid cancers were also described to contain more of the activated (phosphorylated) form of AMP Kinase (AMPK) [147,148]. AMPK plays a central role in the maintenance of redox homeostasis by initiating mitochondrial fission and promoting mitophagy, the removal of damaged mitochondria [149]. Conversely, AMPK is activated by mitochondrial ROS and triggers transcription of mRNAs of antioxidant enzymes [149]. AMPK signaling is also linked to preferential utilization of the glycolytic pathway in diverse cell types, including PTC cells [147,149,150,151]. Metabolic reprogramming of cancer cells is a complex process that is driven both by cell signaling events and thermodynamic rules. Metabolic alterations related to thyroid carcinomas have recently been extensively reviewed [150]. Concerning cell proliferation, AMPK activation leads to cell cycle arrest and diminished cell proliferation in tumor cells in general and also in PTC cells specifically [147,151]. AMPK-mediated signals suppress NOX4 expression and have a negative effect on NOX4-regulated signals in diverse pathologies [152,153]. On the contrary, diminished AMPK activation leads to enhanced NOX4 expression and ROS-mediated cell damage [154]. Figure 4 provides an overview of the various pathways that link NOX4-mediated ROS production to tumor formation. The implication of these NOX4-related signaling mechanisms in diverse forms of thyroid carcinogenesis is currently poorly explored and thus, requires further investigations. A clinically relevant link between NOX4-mediated ROS generation and PTC development is suggested by some GeoProfile datasets that show elevated NOX4 expression in PTC biopsies compared to biopsies obtained from the paired, non-affected thyroid (GDS1732/219773_at and GDS1732/236843_at). Taken together, these data point towards a possible role for NOX4 in PTC proliferation and imply NOX4 as a molecule of interest in pharmaceutical targeting of human thyroid cancers [27,135]. NOX4 is also related to another aspect of tumor propagation, namely in the interaction between the tumor and its microenvironment. Indeed, the complex interactions between immune cells and tumor cells in cancer play a major role in tumor development and subsequent patient outcomes. For example, M2-type tumor-associated macrophages (TAM) contributes to cancer malignant progression. A high density of TAMs positively correlates with tumor invasion, lymph node metastasis and TNM stage, and decreased cancer-related survival in PTC [155]. Furthermore, TAM infiltration was significantly increased in PDTCs and ATCs compared with PTCs. In fact, ATC is characterized by a very dense network of TAMs, representing more than 50% of nucleated cells, which play an important tumor supportive role [156]. Recently, a close association between NOX4 expression and macrophage chemotaxis in patients with non-small cell lung cancer (NSCLC) was found. NOX4 is abundantly expressed in NSCLC and mediates cancer progression. Specifically, tumoral NOX4 has been found to recruit M2 TAM via ROS/PI3K signaling-dependent various cytokine production (including CCL7, IL8, CSF-1 and VEGF-C) to promote non-small cell lung cancer growth [157]. For many other types of cancer cells, NOX4-induced ROS stimulates various inflammatory cytokine expression. Therefore, it is also likely that NOX4 plays a tumor supportive role in thyroid cancer through the recruitment of tumor-associated inflammatory cells including M2 macrophages. However, the interaction between tumoral microenvironment and NOX enzymes, in particular NOX4, remains still largely unidentified. The diverse NOX4-related processes mediating different aspects of thyroid tumorigenesis are depicted in Figure 4. 

## 4. The Thyrocyte Anti-Oxidant Defense System 

ROS and in particular H_2_O_2_ act as important physiological regulators of cellular growth, survival, proliferation and differentiation [158,159,160]. Pathological elevation in H_2_O_2_ concentrations is associated with pro-apoptotic effects manifest in caspase activation, changes in pro-and anti-apoptotic gene expressions and DNA laddering in thyrocytes [161,162,163]. This pro-apoptotic role however, is not univocally considered harmful as apoptosis can also be a protective factor against tumorigenesis [78]. 

**Figure 5:**
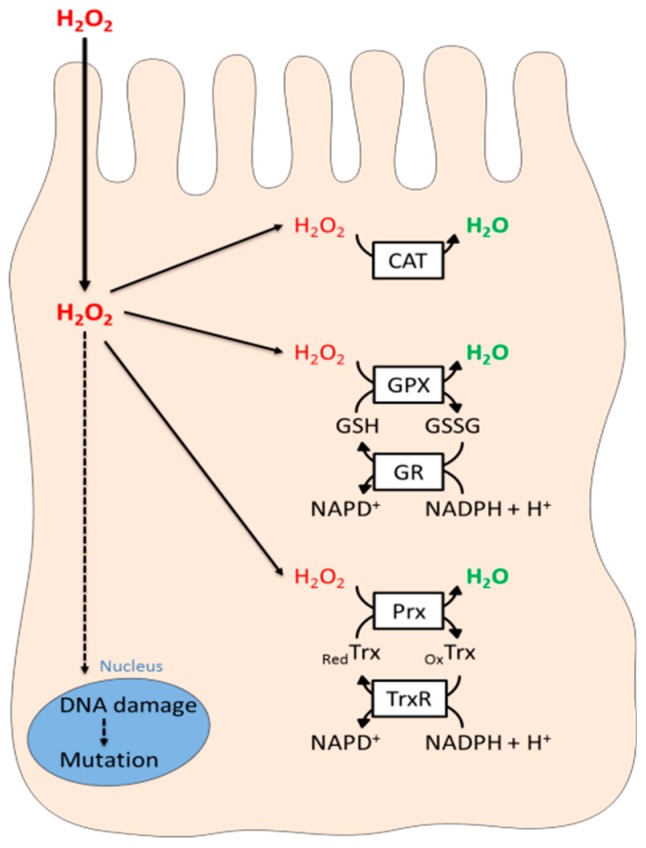
Thyrocyte antioxidant H_2_O_2_ elimination systems. CAT: catalase; GPX: Glutathione peroxidase; GR: Glutathione reductase; GSH and GSSG: reduced and oxidized forms of glutathione; NADP^+^ and NADP + H^+^ oxidized and reduced forms of NADP^+^; Prx: Peroxiredoxin; TrxR: Thioredoxin.

The lifetime of human adult thyrocytes is 7 years thus, precise detection of cellular H_2_O_2_ levels and the development of appropriate gene regulatory and metabolic responses to oxidative insults are crucial to proper thyrocyte function and survival [79,80,161]. The first line of protection lies within the structure of the follicle that provides an enclosure for the H_2_O_2_ contained within its lumen. Indeed, thyrocyte apical membranes present low permeability for H_2_O_2_ and are joined together by tight junctions to prevent paracellular leaking of H_2_O_2_ into the outer follicular space [164]. Within thyrocytes, the redox homeostasis is ensured by anti-oxidant enzymes that promptly degrade any excess H_2_O_2_ that might arise during physiological thyroid hormonogenesis or which might be produced. These antioxidant enzymes encompass catalase, the glutathione peroxidase (Gpx)/glutathione reductase (GR) and the peroxiredoxin (Prx)/thioredoxin reductase (TrxR) systems (Figure 5). Catalase is present in the peroxisomes and is capable of eliminating even toxic levels of H_2_O_2_ by converting it into water and oxygen [165,166]. Catalase has high Km (10 mM) for H_2_O_2_ thus, it is assumed that at patho-physiologically relevant H_2_O_2_ concentrations, the Gpx and TrxR peroxidases are more relevant in H_2_O_2_ elimination [166]. In line with this notion, no thyroid dysfunction was noted in different strains of catalase-deficient mice and in human subjects with acatalasemia [164,167,168,169]. A physiological defensive role for thyroid Gpx and TrxR enzymes was highlighted by data demonstrating a coordinated upregulation of TrxR expression during thyroid hormone synthesis and a direct H_2_O_2_ detoxification role for the extracellularly secreted GPX3 in the thyroid follicle [170,171]. Expression of peroxiredoxin I (Prx I) is stimulated by both TSH and H_2_O_2_ [172]. Overexpression of PrxI promotes elimination of H_2_O_2_ induced by TSH in FRTL-5 cells and protects against H_2_O_2_-induced cellular death [172]. Supra-physiological iodide concentrations inhibit thyroid hormone synthesis and enhance toxic levels of ROS production [173]. In rat PCCI3 thyrocytes, iodide overload induced TrxR transcription leads to a reduction in cellular stress [174]. The implication of oxidative stress in thyroid tumorigenesis is supported by several lines of evidence (reviewed in [175]). Luminal follicular cells situated at the site of H_2_O_2_ production are more exposed to oxidative damage implied by their more prominent staining for 8-oxo-2’-deoxyguanosine (8-OHdG), a mutagenic DNA adduct and a sensitive marker of oxidative DNA damage [176,177]. A role for disturbed redox balance in thyroid carcinogenesis was further substantiated by observations demonstrating that cancerous cells of thyroid tumors presented decreased expression of GPx1 and TrxR1 along with increased amounts of ROS compared to healthy thyrocytes [144]. Interestingly, however, anti-oxidant enzymes might play a bi-directional role in thyroid oxidative stress and tumorigenesis. Indeed, in benign thyroid tumors in rats, *sod3* mRNA levels are increased but are become gradually repressed as cancer cell progress towards a more de-differentiated phenotype [178]. A recent study shed light on the mechanism of this phenomena by demonstrating that mutations in the *RAS* oncogene, one of the commonly found mutations in thyroid malignancies, regulate *sod3* transcription in a biphasic manner [179,180]. Indeed, *sod3* mRNA expression was upregulated at lower *RAS* activation levels (immediate regulatory event), but was completely epigenetically silenced (long-term regulatory event) in cells with higher *RAS* activation [180]. A similar, cellular context dependent regulation was observed for SOD2. In mice, SOD overexpression increased burden of benign tumors but reduced progression of aggressive follicular thyroid carcinoma (FTC). The clinical relevance of these data was supported by findings that reduced *sod2* expression is correlated with poor prognosis for patients with aggressive PTC [181]. 

Taken together, the studies outlined in this review illustrate the complexity of redox homeostasis regulation in thyrocytes and encourage further investigations to explore the individual roles of different pro- and anti-oxidant enzymes in appropriate in vitro and in vivo models of thyroid pathologies.

## 5. Conclusions

The thyroid is a very specific organ with structural and molecular characteristics designed to resist permanent, high level production of oxidative radicals. However, alterations in structural barriers and/or an increase in the ratio between oxidative and anti-oxidative capacity and/or inappropriately sequestered ROS production will lead to thyrocyte oxidative stress with harmful consequences on thyroid hormonogenesis, and eventually, carcinogenesis [74,182]. The exact mechanisms relaying perturbed oxidative homeostasis to different thyroid functional pathologies and tumor development is currently under intensive investigation. In this respect, alterations in DUOX1/2 and NOX4 functions are of emerging interest due to their regulated ROS production and their involvement in diverse aspects of thyroid physiology. NOX-es are increasingly considered as valuable pharmacological targets in the therapy of a variety of diseases [183,184,185,186]. Future studies will undoubtedly provide a better perception of the roles of DUOX and NOX enzymes in thyroid physiopathology. This new knowledge will allow appreciation of DUOX/NOX enzymes as potential novel markers to improve differential diagnosis of thyroid pathologies and will contribute to their recognition as pharmacological targets to contribute to more personalized treatments.
